# Cannabis and Cannabinoids: The Medical Potential of Cannabidiol in Mental and Neurological Disorders

**DOI:** 10.3390/ph19081238

**Published:** 2026-08-06

**Authors:** Răzvan Șolea, Eugenia Șerban, Sabina Florina Călugăr-Solea, Endre Mathe, Nicoleta Mirela Blebea, Gabriel Hancu, Georgeta Serban

**Affiliations:** 1 Doctoral School of Biomedical Sciences, Faculty of Medicine and Pharmacy, University of Oradea, 1 Decembrie 10, 410073 Oradea, Romania; razvanm1989@yahoo.com (R.Ș.); sabi_florina95@yahoo.com (S.F.C.-S.); 2 Department of Environmental Engineering, Faculty of Environmental Protection, University of Oradea, Gen. Magheru 26, 410048 Oradea, Romania; eugeniaserban@yahoo.com; 3 Institute of Nutrition, Faculty of Agriculture and Food Sciences and Environmental Management, University of Debrecen, Boszormenyi 128, HU-4032 Debrecen, Hungary; endre.mathe@agr.unideb.hu; 4 Department of Pharmacology and Pharmacotherapy, Faculty of Pharmacy, “Ovidius” University from Constanța, Căpitan Aviator Al. Șerbănescu 6, 900470 Constanța, Romania; nicoleta.blebea@gmail.com; 5 Department of Pharmaceutical Chemistry, Faculty of Pharmacy, “George Emil Palade” University of Medicine, Pharmacy, Science and Technology of Târgu Mures, Gh Marinescu 32, 540142 Târgu Mures, Romania; gabriel.hancu@umfst.ro; 6 Department of Pharmaceutical and Therapeutic Chemistry, Faculty of Medicine and Pharmacy, University of Oradea, Nicolae Jiga 29, 410028 Oradea, Romania

**Keywords:** *Cannabis sativa*, cannabidiol, anxiety, epilepsy, Alzheimer’s disease, Parkinson’s disease

## Abstract

**Background/Objectives:** Mental and neurological disorders contribute substantially to the global burden of disease, affecting people of all ages and backgrounds. As their prevalence increases with age, their overall impact is expected to grow in the coming decades. Although psychological and pharmacological treatments are available, many patients fail to achieve satisfactory outcomes, underscoring the need for improved therapeutic strategies. *Cannabis sativa* L. has been used for medicinal purposes for centuries, and cannabidiol (CBD) has attracted increasing attention because of its broad therapeutic potential. Scientific studies indicate that CBD may be beneficial in several mental and neurological disorders. **Methods:** A comprehensive literature search was conducted to identify articles investigating the therapeutic potential of CBD and cannabis in selected disorders. **Results:** Evidence from preclinical and clinical studies, together with findings from the broader cannabis literature, indicates that CBD may offer therapeutic benefits in a range of conditions, including Alzheimer’s and Parkinson’s disease, anxiety disorders, and epilepsy. Emerging data also support its potential use as an adjunctive therapy for COVID-19. Current research has improved understanding of the neurobiological mechanisms underlying these disorders and the molecular pathways through which CBD may exert its effects. CBD has demonstrated good tolerability, with predominantly mild adverse effects and a favorable safety profile. **Conclusions:** Despite promising findings, many available studies are preclinical or involve small patient cohorts, and the mechanisms underlying the therapeutic effects of CBD remain incompletely understood. Further well-designed, randomized, controlled, multicenter trials are needed to establish the efficacy and safety of CBD and support its integration into clinical practice.

## 1. Introduction

Mental and neurological disorders comprise a diverse and complex group of highly prevalent conditions, affecting approximately half of the world’s population at some point in life [1]. Collectively, brain disorders, which include mental health conditions, neurological disorders, and cerebrovascular diseases, accounted for more than 15% of global health losses in 2021, as measured by disability-adjusted life years (DALYs). In many cases, their overall burden exceeds that of cancer and cardiovascular diseases, placing a significant strain on healthcare systems and profoundly impacting individuals, families, and society [2]. According to the World Health Organization (WHO), approximately 1.1 billion people, nearly one in seven worldwide, were living with mental health disorders in 2021, with anxiety and depressive disorders among the most common. These conditions carry profound human consequences and impose a substantial economic burden globally. Suicide represents one of the most tragic outcomes, accounting for an estimated 727,000 deaths in 2021 and ranking among the leading causes of death among young people across all countries and socioeconomic groups [3,4]. In addition, sleep disorders represent a significant global health concern, as they are associated with an increased risk of chronic conditions, such as cardiovascular disease, diabetes, and mental health disorders. Beyond their clinical impact, these disorders contribute to cognitive impairment, reduced productivity, a higher incidence of accidents, and increased mortality. The widespread burden of insufficient sleep affects nearly all major public health indicators, including mortality, morbidity, functional performance, and overall quality of life [5]. Furthermore, neurological conditions affect more than 40% of the global population (over 3 billion people) and are responsible for over 11 million deaths annually, according to WHO reports. Almost 85% of this burden occurs in low- and middle-income countries, highlighting substantial global health inequities. As of 2021, the leading contributors to death and disability among neurological disorders include stroke, migraine, Alzheimer’s disease, and epilepsy [6]. Moreover, the 21st century has witnessed the emergence of novel viral infections associated with long-term neurological and cognitive impairment, including COVID-19 and Zika virus infection. These conditions contribute to the growing global burden of neurological disorders, further compounding existing public health challenges [7].

Mental health conditions and neurological disorders are highly prevalent across all countries and communities, affecting individuals of all ages and socioeconomic backgrounds. As populations grow and age, and because the prevalence of many disabling mental and neurological disorders increases markedly with advancing age, the overall burden of these conditions is expected to rise substantially. Although effective psychological therapies are available and pharmacological treatments may also be used, these interventions do not always achieve optimal outcomes for all patients. This underscores the urgent need for greater investment in mental health care and research. Advancing scientific understanding is essential for developing more effective strategies for prevention, early intervention, and treatment [4].

**Figure 1:**
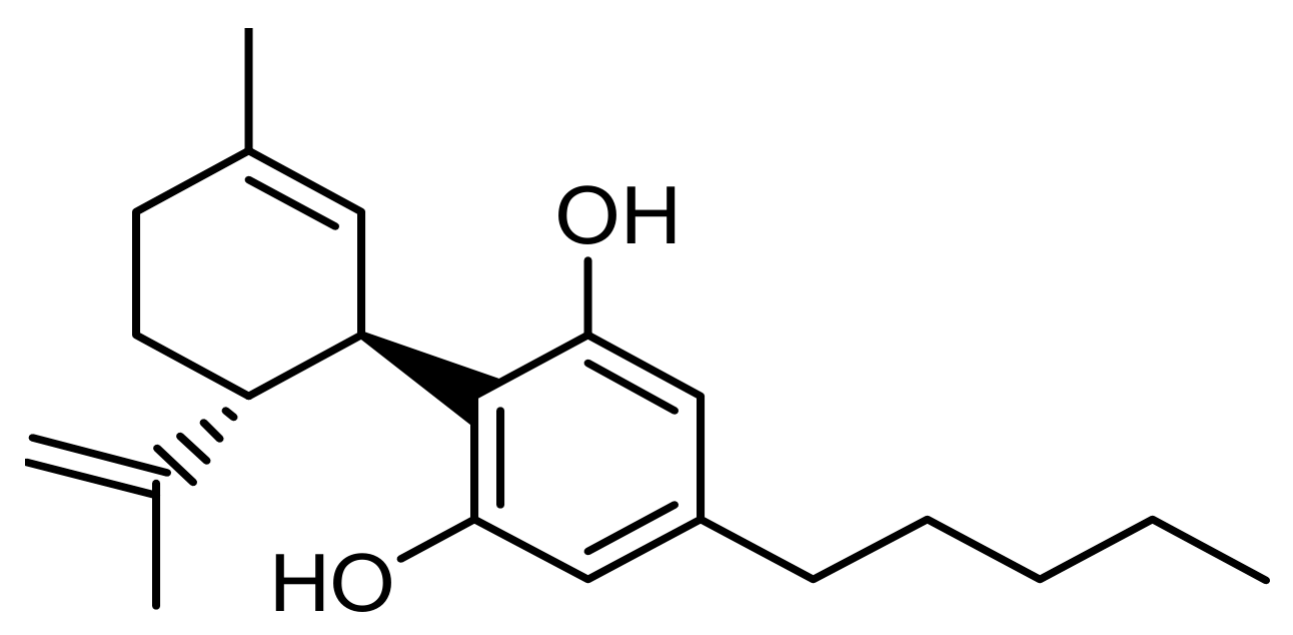
Chemical structure of cannabidiol.

*Cannabis sativa* L. is an annual flowering plant belonging to the Cannabaceae family, which has been used since ancient times as a source of textile fibers, food, oil, and traditional medicine, as well as for religious rituals [8]. Although its origin is uncertain, it is assumed to have originated in Central Asia (Altai, Caucasus, Himalayan Mountains, etc.), and recent fossil pollen studies suggest the northeastern Tibetan Plateau as its centre of origin [9,10]. Following the dispersal of its seeds along the Silk Road, the species adapted to diverse climatic conditions and is now distributed worldwide [11,12], [13] (pp. 11–12). Due to extensive crossbreeding, it is now accepted that the genus Cannabis consists of a single species (*Cannabis sativa* L.), which is divided into several subspecies. Three main subspecies are distributed worldwide and differ in chemical and morphological characteristics: *C. sativa* subsp. *sativa* (hemp, <0.3% Δ^9^-tetrahydrocannabinol (THC)) cultivated for fibre, *C. sativa* subsp. *indica* (marijuana, >0.3% Δ^9^-THC), which exhibits psychoactive properties, and *C. sativa* subsp. *ruderalis* with intermediate characteristics, used to create hybrids resistant to various climatic conditions [8,12,14]. Cannabinoids derived from the hemp plant have considerable medical potential and are currently being investigated for their analgesic, anti-inflammatory, anticancer or immunomodulatory properties [15,16,17,18]. Cannabidiol (CBD) (Figure 1), one of the major cannabinoids found in the Cannabis plant, is considered to have a more favourable benefit-to-risk profile than other cannabinoids. This is largely due to its lack of psychoactive effects, unlike THC, which produces the characteristic “high”, typically described as a state of euphoria and altered perception [19].

Like other biologically active natural compounds [20,21,22], CBD is currently being intensively investigated. Numerous recent studies have highlighted the therapeutic potential of CBD in the management and treatment of a wide range of acute and chronic conditions, including central nervous system disorders [15,16,23]. In addition, several studies have reported synergistic interactions between CBD and other bioactive compounds, such as bioflavonoids, which may enhance therapeutic efficacy [19,23]. These findings support the safety and clinical applicability of CBD and further suggest its potential as a therapeutic agent [15,16,19,23].

The objective of this review is to critically evaluate the current evidence regarding the therapeutic potential of CBD in major mental and neurological disorders. Specifically, this review addresses the following research question: What is the current level of evidence supporting the therapeutic use of CBD across different mental and neurological disorders, and what are the main gaps that need to be addressed before broader clinical implementation can be recommended? The article presents a narrative review of preclinical studies and clinical reports evaluating the therapeutic efficacy, mechanism of action, and safety profile of CBD in selected mental and neurological conditions. Furthermore, the review identifies current gaps in knowledge and outlines key priorities and potential directions for future research.

Unlike previous reviews focusing on individual mental or neurological disorders, the present review provides an integrated evaluation of the therapeutic potential of CBD across multiple disorders. Emphasis is placed on comparing the current level of evidence, discussing shared molecular mechanisms, identifying methodological limitations, and highlighting common research gaps that may guide future clinical investigations.

## 2. Medical History of Cannabis

The Cannabis plant has been used for medicinal purposes for thousands of years, with therapeutic indications mentioned in the medical texts of ancient civilizations. These manuscripts reported the use of cannabis to treat a wide range of health problems. In ancient China (2700 BC), cannabis was used for rheumatic pain, constipation, female reproductive disorders, and malaria, and later as an anaesthetic in surgical interventions [24,25,26,27]. In ancient India (2000–1000 BC), cannabis was used both as a medicine and as a recreational drug, its psychoactive effects being well known [8,9,10]. In Ayurvedic medicine, cannabis was believed to balance the body’s functions, and it was used for various types of pain (analgesic), anxiety and insomnia (tranquilizer/hypnotic), gastrointestinal disorders (digestive, antiemetic and appetite stimulant), muscle contractions (antispastic), as well as to induce a feeling of euphoria [25,26,28,29]. In ancient Egypt (1550 BC), the Ebers Papyrus describes cannabis as a remedy for inflammation and glaucoma, while topical preparations were used to treat inflamed eyes and haemorrhoids [10], [13] (p. 17), [26]. In the Assyrian Empire (900 BC), cannabis was used for the treatment of inflammation, depression, arthritis, kidney disease, or gynaecological disorders, and in religious rituals [25,26,30]. Cannabis use in Europe may have predated the Christian Era. Archaeological evidence suggests that ancient people living in what is now Romania used hemp seeds in funeral rituals over 5000 years ago [13] (p. 13), [31]. Historical sources describing cannabis in Ancient Greece and Rome (500 BC–500 CE) suggest that it was used in funeral ceremonies, as well as for the treatment of pain and inflammation (gout, arthritis, earaches) and as an antiparasitic treatment for tapeworm infections [13] (p. 18), [25,26,28]. The Greek physician Dioscorides was aware of the psychotropic properties of the plant, and Galen highlighted the effects of cannabis abuse on the brain [8]. Medieval Islamic medical manuscripts (7th–13th centuries) mention *C. sativa* extract as having several medicinal effects, such as sedative, antiepileptic, analgesic, diuretic, anti-inflammatory, antiemetic, antipyretic, or antiparasitic effects [25,28,32], while Persian medical texts from the 12th century mention the use of boiled roots and Cannabis leaves to treat fever, inflammation and dermal infections [33]. Cannabis use in Africa has been associated with snakebites, childbirth, malaria, and fever and is thought to date back to at least the 15th century [25,34]. Although there is little information about the origin of cannabis in the New World, evidence of cannabis textiles has been discovered among pre-Columbian Native American civilizations in the Great Lakes area and the Mississippi Valley [8]. However, it is assumed that cannabis arrived in Brazil with enslaved people from Angola in the 16th century, where it was used both in religious practices and for medical purposes (including toothache and uterine spasms) [25,34]. The Irish physician William Brooke O’Shaughnessy reintroduced cannabis to Western medicine in 1838, after studying its use in India and reporting significant improvement in patients with severe chronic arthritis. Cannabis preparations were used primarily for muscle spasms, rheumatism, convulsions, and nausea [25,26,35,36].

## 3. The Endocannabinoid System and CBD

The endocannabinoid system (ECS) is a complex lipid network comprising the endocannabinoid receptors CB1 and CB2, endogenous ligands called endocannabinoids, and the enzymes responsible for the synthesis and degradation of endocannabinoids. As a neuromodulatory system, the ECS plays a critical role in maintaining and modulating various physiological processes involving the CNS. These endogenous compounds are synthesized in mammals in response to increased intracellular calcium levels [37]. The mechanism of action of cannabinoids is still under intensive investigation. In general, cannabinoids exert their effects through interactions with *CB1 and CB2 receptors* in the ECS, resulting in immunomodulatory, neuroprotective, antioxidant, analgesic, anxiolytic, and antiepileptic effects [38]; through interactions with *serotonin (5-HT) receptors*, leading to antidepressant and anxiolytic effects [39,40]; and *vanilloid-type TRPV receptors,* resulting in reduced motor function, euphoria, analgesia, and immunomodulation [41,42].

CBD is the best-tolerated cannabinoid found in the cannabis plant and acts by modulating endocannabinoid signalling indirectly and inhibiting the hydrolysis of the endocannabinoid anandamide. CBD is also a partial CB2 agonist with no psychotropic side effects and a negative allosteric modulator with a very low affinity for both cannabinoid receptors, which indirectly activates CB1 with a lower affinity than THC and 2-arachidonoylglycerol (2-AG). This may enhance endogenous cannabinoid tone, contributing to CBD’s pharmacological effects [43,44]. Beyond the endocannabinoid system, CBD interacts with multiple receptor systems implicated in emotional regulation, such as the 5-HT1A serotonin receptors [39]. In addition, CBD modulates nuclear receptors peroxisome proliferator-activated receptor gamma (PPARγ), the orphan receptors GPR55 and GPR18, and the μ- and δ-opioid receptors [40,45], as well as activates the TRPV1 and TRPV2 receptors, and these interactions contribute to the multifaceted effects of CBD, including on neural circuits involved in stress, anxiety, and affective regulation [43,44]. GPR18 and GPR55 receptors are considered potential targets for various phytocannabinoids, endocannabinoids, and synthetic compounds. These receptors participate in the regulation of cellular signalling pathways involved in various metabolic disorders and are localized both in the CNS and in the peripheral system, including the brain and vascular system. As for the GPR18 receptor, evidence suggests that it may influence inflammatory and immune processes by regulating mechanisms such as leukocyte migration and programmed death. CBD has been reported to modulate epigenetic regulatory mechanisms, including the activity and expression of DNA methyltransferases (DNMTs), potentially leading to alterations in DNA methylation patterns [46]. Additionally, CBD exposure has been associated with alterations in histone modification profiles and gene expression [47]. Furthermore, CBD has been shown to influence non-coding RNAs, particularly microRNAs, which are key regulators of post-transcriptional gene expression [47]. Notably, most of these findings originate from in vitro and animal models, and further studies are required to establish their translational relevance in humans [46].

The ability of CBD to modulate DNA methylation patterns and to engage serotonergic pathways via the 5-HT1A receptors may contribute to its antidepressant-like effects observed in preclinical models [40,48]. In addition, CBD’s ability to modulate the levels of DNA methyltransferases (DNMT1 and DNMT3a) is of particular interest, since these enzymes have been reported to exhibit altered regulation in mood disorders, including bipolar disorder, suggesting the potential of CBD in the treatment of bipolar disorder. However, the functional significance of these alterations remains incompletely understood, and their direct therapeutic implications are yet to be established [40,49]. Furthermore, dysregulation of inflammation-related microRNAs such as miR-146a and miR-155 has been documented in depressive disorders and linked to neuroinflammatory pathways [50]. Preclinical studies indicate that CBD can modulate microRNA expression, suggesting a potential role in modulating neuroinflammatory pathways linked to depressive-like phenotypes, although direct evidence in humans remains limited [40,48].

CBD is a highly lipophilic compound with low and variable oral bioavailability, due to extensive first-pass metabolism. Following oral administration, peak plasma concentrations are reached within 2–5 h, although absorption is influenced by formulation and food intake, with high-fat meals substantially increasing systemic exposure. CBD undergoes extensive hepatic metabolization, mainly by cytochrome P450 enzymes, particularly CYP2C19 and CYP3A4, generating several hydroxylated and carboxylated metabolites that are subsequently eliminated in the faeces and urine. Elimination half-life varies considerably depending on the route of administration and duration of treatment. Because CBD inhibits several CYP enzymes, clinically relevant drug–drug interactions may occur, especially in patients receiving antiepileptic drugs or other medications with narrow therapeutic indices. These pharmacokinetic characteristics likely contribute to the variability in dosing regimens and therapeutic responses reported across clinical studies [13] (pp. 75–77, 157–158), [43,44].

The pharmacological characteristics of CBD provide a strong biological rationale for its investigation in a broad spectrum of mental and neurological disorders. Unlike many conventional agents that act through a single molecular target, CBD exhibits a complex pharmacological profile involving modulation of the endocannabinoid system together with interactions with multiple receptor systems and intracellular signalling pathways. These pleiotropic actions are thought to contribute to its anti-inflammatory, antioxidant, neuroprotective, anxiolytic, anticonvulsant, and neuromodulatory effects observed in both experimental and clinical studies. At the same time, the therapeutic response to CBD may be influenced by its variable pharmacokinetic profile, formulation, route of administration, dosage regimen, treatment duration, and potential drug–drug interactions. Consequently, these pharmacological considerations should be considered when interpreting the heterogeneous findings reported across different mental and neurological disorders and when comparing the results of studies [13] (pp. 75–77), [39,40,41].

## 4. Therapeutic Potential of CBD in Anxiety, Mood and Sleep Disorders

The therapeutic potential of CBD in anxiety, mood, and sleep disorders has been investigated extensively in both preclinical models and clinical studies. Experimental studies have provided important insights into the neurobiological mechanisms underlying its effects, while clinical investigations have evaluated its efficacy, safety, and tolerability across different patient populations.

The main clinical studies investigating the therapeutic potential of CBD in anxiety and sleep disorders are summarized in Table 1.

Several preclinical studies [44] support CBD as a potential therapeutic agent for anxiety and mood disorders. The available data derive primarily from rodent models (rats and mice) and indicate that CBD produces consistent anxiolytic and antidepressant-like effects across validated behavioural paradigms. Anxiety-related behaviours were assessed using the elevated plus maze (EPM), Vogel conflict test (VCT), and social interaction tests, whereas depressive-like phenotypes were evaluated using the forced swim test (FST), tail suspension test (TST), and olfactory bulbectomy (OBX) model, which are widely accepted translational measures of affective states. CBD exhibits a multimodal mechanism of action, with serotonergic modulation, particularly via 5-HT1A receptors, being considered the principal mechanism. Pharmacological blockade of 5-HT1A receptors attenuates CBD’s behavioural effects, supporting their central role. Additional mechanisms include indirect enhancement of endocannabinoid signalling and emerging epigenetic effects, such as changes in DNA methylation and microRNA expression, suggesting effects of gene expression and neuroplasticity relevant to long-term emotional adaptation. Doses and routes of administration varied considerably across studies. Systemic intraperitoneal administration was most common, with anxiolytic effects typically observed at acute doses of 2.5 mg/kg, while lower doses (≈1 mg/kg) were effective in some cases. Higher doses (up to 200 mg/kg) were tested in depressive models or in combination with standard antidepressants. Behavioural outcomes often follow an inverted U-shaped dose–response curve, with moderate doses producing optimal efficacy. Overall, CBD consistently reduced anxiety- and depressive-like behaviours across models, supporting its potential as a therapeutic candidate [44].

A growing body of evidence suggests that several psychiatric disorders, including major depressive disorder, anxiety, post-traumatic stress disorder or schizophrenia, are associated with alterations in the ECS [51,52]. Compared with healthy controls, these alterations may include changes in cannabinoid receptor availability, particularly of CB1 receptors, as well as variations in circulating or central levels of endogenous cannabinoids such as anandamide (AEA) and 2-arachidonoylglycerol (2-AG) [52,53]. In a clinical study of medication-free patients with major depressive disorder, Hill et al. [54] observed significantly reduced serum levels of 2-AG compared to healthy controls and a negative correlation between 2-AG concentrations and the duration of depressive episodes. Although serum AEA levels were not significantly altered across groups, AEA concentrations were inversely correlated with Hamilton Anxiety Rating Scale scores. Together, these results indicate an association between peripheral endocannabinoid signalling and the chronicity of depressive symptoms, as well as the severity of anxiety symptoms [54]. These data suggest that ECS is involved in cognitive and emotional processes in the brain through its ability to modulate hippocampal neurogenesis. An imbalance in the proper functioning of the ECS may contribute to the development and severity of neuropsychiatric diseases [55]. However, findings are heterogeneous and depend on factors such as the specific disorder, brain region examined, disease stage, and methodological approach [52,56].

Several studies have investigated the effects of CBD in social anxiety disorder (SAD) and sleep disorders across different age groups and experimental paradigms. In a single-dose, double-blind, placebo-controlled study, Crippa et al. administered 400 mg of oral CBD to adults prior to a simulated public speaking test (SPST). CBD significantly reduced anxiety, as measured by subjective social anxiety scores, compared with placebo, and modulated regional cerebral blood flow in limbic and paralimbic regions, including the amygdala, left parahippocampal gyrus, hippocampus, and anterior cingulate cortex [57]. In a similar study, Bergamaschi et al. demonstrated that a single oral dose of 600 mg of CBD administered to adults, approximately 90 min before the SPST, significantly attenuated anxiety, cognitive impairment, and discomfort as assessed using the Visual Analogue Mood Scale (VAMS) and the Negative Self-Statement Scale (SSPS-N), without affecting physiological parameters such as heart rate or blood pressure. In contrast, placebo-treated patients exhibited higher anxiety and negative self-evaluations during the test, indicating that CBD can acutely reduce experimentally induced social anxiety symptoms, primarily by reducing the subjective components of anxiety and negative self-perception during public speaking [58].

Furthermore, the acute anxiolytic effects of CBD in humans were demonstrated in a randomized, double-blind, placebo-controlled study by Linares et al. involving healthy male participants undergoing a SPST. The authors identified a clear inverted U-shaped dose–response curve, with a single oral dose of 300 mg of CBD producing a statistically significant reduction in subjective anxiety compared with placebo, while lower (150 mg CBD) and higher (600 mg CBD) doses were ineffective. Quantitatively, the 300 mg dose reduced the maximum anxiety scores (VAMS) by approximately 10–15 points compared to placebo, corresponding to an estimated eightfold decrease during the speech phase, which represents the period of maximum induced anxiety. Notably, CBD did not significantly alter physiological measurements such as heart rate or blood pressure, further suggesting that its anxiolytic effects are primarily mediated at the subjective level. These findings provide controlled human evidence for dose-dependent anxiolysis with CBD, while also highlighting non-linear pharmacodynamics in humans and a narrow therapeutic window for acute administration [59]. Extending these observations to adolescents, Masataka conducted a 4-week trial of daily 300 mg CBD in Japanese adolescents, resulting in a significant reduction in anxiety, as measured by the Fear of Negative Evaluation (FNE) scale and the Liebowitz Social Anxiety Scale (LSAS) during public speaking, compared with only minor changes in the placebo group [60]. These findings suggest that CBD may reduce both evaluative and performance-related anxiety in adolescents, highlighting its potential as a therapeutic option for paediatric social anxiety.

Traditional medical systems historically incorporated *Cannabis sativa*, including hemp-derived preparations, for conditions associated with restlessness and sleep disturbance. In recent years, there has been growing clinical and research interest in the use of CBD for sleep disorders, with multiple clinical studies examining its effects on sleep quality and different dosing and administration strategies [61]. Shannon et al. conducted a large retrospective chart review of 72 adults treated with oral CBD (typical dose of 25 mg/day) as an adjunct to usual care for anxiety and sleep complaints. Anxiety and sleep improved in most patients, and these improvements were maintained over time. During the first month of CBD administration, 79.2% of participants showed clinically documented reductions in anxiety severity, and 66.7% demonstrated improvements in sleep quality. Overall, the results suggested that low doses of CBD, administered chronically, may help reduce anxiety with possible but less consistent effects on sleep. These preliminary findings support the potential use of CBD in anxiety and sleep disorders [15]. In a cross-sectional online survey of 387 current or former CBD users, Moltke et al. reported that anxiety (42.6%), sleep disturbances (42.5%), stress (37%), and general health and well-being (36.5%) were among the most cited reasons for using CBD. Most respondents reported using relatively low daily doses (less than 50 mg/day), with the main route of administration being sublingual (72.6%). Among individuals using CBD for specific symptoms, a substantial proportion reported perceived improvements in anxiety, stress and sleep problems following CBD use [62]. To evaluate the effects of low-dose, subchronic CBD treatment, Winkler, Meis, and Hermann conducted a randomized controlled trial involving 180 university students experiencing elevated stress and at risk of depression. Participants were assigned to receive CBD oil, placebo oil, or no treatment, with CBD doses gradually titrated from 0.02 mg/kg to 1 mg/kg body weight over 30 days. Both the CBD and placebo groups showed statistically significant reductions in perceived stress, depressive symptoms, and somatization relative to the untreated control group, with small to moderate effect sizes. However, no significant differences were observed between the CBD and placebo groups, indicating no evidence of drug-specific effects beyond placebo. Furthermore, no significant improvements were detected in anxiety, sleep quality, or general well-being across groups. Although the CBD group showed reductions in measures such as the Perceived Stress Scale (PSS-10 decreased from 26.25 to 18.71) and the Patient Health Questionnaire (PHQ-15 decreased from 11.25 to 7.12), these changes suggest modest symptom improvement and are likely of limited clinical relevance at the individual level. Therefore, these findings suggest that the observed improvements were largely attributable to placebo effects rather than to the pharmacological effects of low-dose CBD in this population [63].

**Table 1:** Summary of clinical studies investigating the therapeutic potential of CBD in anxiety and sleep disorders.

Study	Study Design	Subjects	CBD Regimen	Main Findings	Limitations
Crippa et al. (2011) [57]	Randomized, double-blind, placebo-controlled crossover study	10 treatment-naïve patients with SAD; 20–33 years old	400 mg orally; single dose	Reduced subjective anxiety during the SPST (SAD score 30.8 ± 7.7 for CBD vs. 42.1 ± 10.3 for placebo). SPECT imaging demonstrated changes in regional cerebral blood flow within limbic and paralimbic brain areas associated with anxiety modulation.	Small sample size; acute administration only.
Bergamaschi et al. (2011) [58]	Randomized, double-blind, placebo-controlled trial	24 patients with SAD; 18–35 years old	600 mg orally; single dose before SPST	Significantly reduced anxiety, cognitive impairment, and discomfort during public speaking. No clinically relevant cardiovascular changes or serious adverse effects. The effect of CBD is mainly subjective/cognitive, rather than autonomic.	Small sample size; short-term study; evaluated only acute effects.
Linares et al. (2019) [59]	Randomized, double-blind, placebo-controlled trial	57 healthy male adult volunteers	150 mg (*n* = 15), 300 mg (*n* = 15), or 600 mg (*n* = 12) orally; single dose	Demonstrated an inverted U-shaped dose–response relationship: 150 mg, insufficient effect; 300 mg, optimal anxiolytic effect; 600 mg, loss of effect. No clinically relevant cardiovascular changes.	Conducted in healthy volunteers rather than patients with anxiety disorders.
Masataka et al. (2019) [60]	Randomized, double-blind, placebo-controlled trial	37 Japanese adolescents with SAD; 18–19 years old	300 mg/day orally for 4 weeks	Significantly reduced FNE and LSAS scores; improved social anxiety symptoms in adolescents. CBD was well tolerated throughout treatment.	Single-center study with a relatively small sample size.
Shannon et al. (2019) [15]	Retrospective case series	72 adults presenting with anxiety (*n* = 47) and/or sleep disorders (*n* = 25); 18–72 years old	Usually 25 mg/day orally for 1–3 months; dose adjusted individually (50, 75 or 175 mg/day)	Anxiety scores improved in 79.2% of patients during the first month. Sleep scores improved in 66.7% of patients during the first month. CBD was generally well tolerated. Side effects were rare and mild. CBD shows promising potential for anxiety disorders and modest/good sleep benefits.	Retrospective design; absence of placebo control; heterogeneous patient population.
Moltke et al. (2021) [62]	Cross-sectional observational survey (online questions)	387 adult CBD users with anxiety, sleep disorders or stress; 25–54 years old	Variable oral (32.8%) or sublingual (72.6%) doses (most ≤50 mg/day)	Most participants reported improvements in anxiety (141/163 respondents), stress (130/141 respondents), and sleep quality (73/124 respondents). Adverse effects were generally mild and infrequent.	Self-reported outcomes; observational design; causality cannot be established.

CBD, cannabidiol; FNE, Fear of Negative Evaluation; LSAS, Liebowitz Social Anxiety Scale; SAD, social anxiety disorder; SPST, Simulated Public Speaking Test; SPECT, single-photon emission computed tomography.

In recent years, the COVID-19 pandemic has been associated with a substantial increase in psychological distress worldwide, with elevated rates of anxiety, depression, post-traumatic stress symptoms (PTS), and post-traumatic stress disorder (PTSD) reported both in the general population and among high-risk groups, including healthcare workers and survivors of COVID-19 infection. Epidemiological studies conducted across multiple countries consistently have highlighted the significant burden of pandemic-related mental health sequelae, indicating an urgent need for additional therapeutic strategies. Conventional pharmacological and psychological interventions for anxiety and stress-related disorders remain limited by delayed onset of action, incomplete or variable efficacy, relapse following discontinuation, and adverse effects, prompting interest in alternative or adjunctive treatments. Consequently, CBD has attracted considerable interest as a potential therapeutic option for anxiety disorders. Preclinical and early clinical evidence suggests that CBD modulates several neurobiological systems implicated in stress and anxiety regulation, including the facilitation of 5-HT1A receptor signalling and activation of TRPV1 channels, modulation of the endocannabinoid system, and attenuation of inflammatory and oxidative processes associated with mood and stress dysregulation [63,64,65]. In this context, Crippa et al. conducted the BONSAI (Burnout and Distress Study) study, a prospective, randomized, open-label clinical trial that evaluated the efficacy and safety of adjunctive CBD in reducing burnout and emotional distress among healthcare workers during the COVID-19 pandemic. The results demonstrated that the addition of oral CBD (300 mg/day, for 28 days) to standard therapy was associated with significant reductions in emotional exhaustion, anxiety, and depressive symptoms, suggesting a beneficial effect on key dimensions of burnout in this high-risk population. Burnout among healthcare workers represents a major challenge for healthcare systems because of its direct impact on the quality of patient care, highlighting the need for larger studies and careful clinical monitoring to determine whether CBD may have a role as a therapeutic strategy for healthcare professionals exposed to pandemic-related stressors [66]. On this basis, O’Sullivan et al. [65] proposed that CBD could represent a potential therapeutic option for anxiety disorders associated with the COVID-19 pandemic. The authors placed this hypothesis in the context of a marked global increase in symptoms of anxiety, depression, and post-traumatic stress associated with pandemic-related stressors, including social isolation, health concerns, and economic instability. Based on preclinical, clinical, and observational evidence, the study highlights the anxiolytic, anti-inflammatory, and sleep-modulating properties of CBD and reports that acute oral administration of CBD, typically at doses ranging from 300 to 800 mg, reduces anxiety in a variety of populations, including healthy volunteers exposed to experimental stress paradigms, patients with social anxiety disorder, and individuals at clinically elevated risk for psychiatric disorders [65]. Across these studies, CBD was generally well tolerated and exhibited a favourable safety profile compared to many conventional anxiolytic drugs. However, despite these encouraging findings, direct evidence supporting the use of CBD specifically for anxiety or PTSD associated with COVID-19 remains limited, and adequately designed clinical trials are still needed in populations affected by the pandemic.

The anti-inflammatory properties of CBD are considered to be beneficial in viral infections where the host’s inflammatory response is pathogenic and not related to clearance and recovery. They can function as immunomodulators in the treatment of COVID-19. CBD can cause apoptosis in mammalian cells by affecting TRPV1 receptors and increasing reactive oxygen species (ROS), which is a critical response during viral infections [63,64,65]. Several enzymes, ion channels, transporters, and protein receptors are affected by this chemical. For example, it is a PPARγ agonist that belongs to the nuclear hormone receptor family. PPAR-γ is crucial for the regulation of cytokine over-secretion, and PPAR-γ agonism in alveolar macrophages can substantially reduce lung inflammation. CBD can reduce the generation of cytokines that induce lung damage and prevent the development of pulmonary fibrosis caused by COVID-19 in this way [66]. CBD can also stop viruses from reproducing after they have entered cells. As a result, it should be effective against viral strains with mutant SARS-CoV2 spike proteins.

Collectively, these studies indicate that CBD exerts both acute and potential subchronic/chronic anxiolytic effects across age groups, possibly through modulation of the limbic brain regions and serotonergic/endocannabinoid systems. Acute effects have been observed in controlled settings, often at moderate to high doses, while preliminary evidence suggests that chronic use may provide incremental benefits at low or variable doses. Current clinical evidence for anxiety disorders is encouraging but remains limited, as it is based primarily on small randomized controlled trials and pilot clinical studies. Similarly, the available evidence for sleep disorders is still preliminary, owing to the limited number of controlled clinical studies and the heterogeneity of study designs, treatment protocols, and outcome measures. However, as evidence remains limited, controlled clinical studies are necessary to confirm efficacy and safety, establish optimal dosing, and determine long-term applicability of CBD in humans.

## 5. The Efficacy of CBD in Epilepsy Treatment

Epilepsy is one of the most common chronic neurological disorders and remains a major global health challenge because of its high prevalence, heterogeneous clinical presentation, and substantial impact on quality of life. Although numerous antiseizure medications are available, approximately one-third of patients continue to experience seizures despite appropriate pharmacological treatment, highlighting the need for alternative therapeutic approaches [67]. The medicinal use of cannabis dates to antiquity; ancient Mesopotamian cuneiform tablets, including the Kouyunjik texts, have been interpreted as describing cannabis-based remedies for convulsive or seizure-like disorders. Interest in CBD has increased considerably over the past decade following encouraging preclinical findings and the subsequent demonstration of clinical efficacy in several forms of drug-resistant epilepsy [68].

The main clinical studies investigating the therapeutic potential of CBD in epilepsy are summarized in Table 2.

Falenski et al. examined changes in cannabinoid CB1 receptor expression following pilocarpine-induced status epilepticus (SE). Within one week after SE, a marked decrease in CB1 receptor immunoreactivity was observed throughout the hippocampus. One month after SE, which corresponds to the onset of spontaneous recurrent seizures in this model, CB1 receptor expression exhibited a layer-specific redistribution. These findings suggest that CB1 receptors undergo dynamic reorganization during epileptogenesis, rather than undergoing uniform downregulation [69]. CB1 receptors play a crucial modulatory role in neuronal excitability and seizure activity. Endocannabinoid activation of presynaptic CB1 receptors normally suppresses the release of excitatory neurotransmitters, thereby reducing neuronal hyperexcitability and limiting the duration and frequency of seizures. Redistribution of CB1 receptor expression has been linked to altered seizure susceptibility in both animal models and human epileptic tissue, suggesting that CB1 receptor dysregulation contributes to epileptogenesis. These findings identify the endocannabinoid system as a potential therapeutic target in epilepsy, especially for drug-resistant epilepsy [70].

CBD has been investigated extensively as a potential therapy for treatment-resistant epilepsy over the past five decades [71]. Initial preclinical studies conducted by Carlini et al. in the 1970s demonstrated that CBD significantly reduced the incidence of seizures in audiogenic seizure-susceptible rodents. Specifically, seizure frequency decreased from 60% in untreated animals to 10% in CBD-treated groups [72]. Early human trials, such as those conducted by Cunha et al., provided preliminary evidence for the anticonvulsant effects of CBD (200–300 mg/day, orally) in a small cohort of patients (*n* = 4) with refractory epilepsy [73]. Open-label, multicenter, expanded-access clinical trials conducted in 2016 by Devinsky et al. further confirmed the safety, tolerability, and potential efficacy of adjunctive CBD in a larger cohort of children and young adults with severe epilepsy, including Dravet syndrome (DS, ≈20% patients), Lennox–Gastaut syndrome (LGS, ≈19% patients), and other drug-resistant epilepsy (DRE) syndromes for whom conventional antiepileptic drugs failed to control seizures [74].

Building on these observations, subsequent randomized, placebo-controlled clinical trials have provided substantial evidence supporting the efficacy of CBD in specific treatment-resistant epilepsy syndromes. In the pivotal phase III trial published in 2017, Devinsky et al. demonstrated that adjunctive CBD administration significantly reduced seizure frequency in patients with DS over a 14-week treatment period compared with placebo, while improving caregiver-reported global outcomes [75]. Similarly, randomized phase III trials published in 2018 [76,77] showed that CBD produced a significant reduction in seizure frequency in patients with LGS compared to standard therapy alone. Taken together, these studies provide robust evidence that CBD confers a clinically meaningful, though partial, reduction in seizure burden in two highly pharmacoresistant childhood epilepsies, Dravet syndrome and Lennox–Gastaut syndrome. However, treatment effects responses varied among patients, seizure freedom was uncommon, and adverse events, including somnolence, gastrointestinal symptoms, and transaminase elevations, were frequent, underscoring the need for careful monitoring and realistic expectations regarding treatment benefit [75,76,77,78]. Despite these limitations, the consistent efficacy observed in controlled trials, together with a generally manageable safety profile, supported the 2018 approval by the U.S. Food and Drug Administration (FDA) of pharmaceutical-grade CBD (Epidiolex) as an adjunctive treatment for seizures in patients aged ≥2 years with Dravet and Lennox–Gastaut syndromes, and tuberous sclerosis complex. This approval represented an important, albeit incremental, advance in cannabinoid-based therapies for epilepsy [79]. In 2019, the European Commission authorized the highly purified CBD formulation (Epidyolex) for Dravet and Lennox–Gastaut syndromes in the EU following a positive opinion from the European Medicines Agency (EMA), further supporting its regulatory acceptance in major jurisdictions [80].

In the following years, various studies have examined CBD or cannabis extracts as treatments for epilepsy [81,82,83,84]. Some observational evidence suggests that CBD-rich whole-plant cannabis extracts may demonstrate higher therapeutic efficacy than purified CBD preparations [85]. Accordingly, McCoy et al. studied a cannabis extract (TIL-TC150 containing 100 mg/mL CBD and 2 mg/mL THC) and reported that it was generally well tolerated and associated with substantial reductions in seizure frequency, improved EEG biomarkers, and improved quality of life in paediatric patients with DS and DRE. These results provide preliminary data on the dosing, safety, and efficacy of cannabinoid preparations containing both CBD and low levels of THC, highlighting the need for future randomized, placebo-controlled studies to evaluate the anticonvulsant efficacy of these extracts and to distinguish their effects from those of concomitant antiepileptic therapy and placebo effects [86].

**Table 2:** Summary of clinical studies investigating the therapeutic potential of CBD in epilepsy.

Study	Study Design	Subjects	CBD Regimen	Main Findings	Limitations
Devinsky et al. (2016) [74]	Open-label, multicenter expanded-access clinical trial	214 patients with severe refractory epilepsy (DS ≈ 20%, LGS ≈ 19%); 1–30 years old	Oral CBD, 2–5 mg/kg/day titrated to 25–50 mg/kg/day for 12 weeks	Median reduction in motor seizures of 36.5%. 39% of patients achieved >50% seizure reduction. CBD demonstrated acceptable tolerability and provided preliminary evidence of efficacy supporting subsequent randomized trials.	Open-label design; no placebo control; heterogeneous patient population.
Devinsky et al. (2017) [75]	Randomized, double-blind, placebo-controlled, multicenter trial	120 patients with DS and DRE; 2–18 years old	Oral CBD, 20 mg/kg/day for 14 weeks	Median seizure reduction of 39%. 43% of patients achieved ≥50% seizure reduction (vs. 27% with placebo). 5% of patients became seizure-free. Manageable adverse events. First high-quality RCT.	Limited to Dravet syndrome; relatively short treatment period.
Devinsky et al. (2018) [76]	Randomized, double-blind, placebo-controlled trial	225 patients with LGS; 2–55 years old	Oral CBD, 10–20 mg/kg/day for 14 weeks	Drop seizures decreased by 37–42% versus 17% with placebo. 36–43% of patients achieved ≥50% seizure reduction (vs. 14–27% placebo). CBD was generally well tolerated.	Limited to Lennox–Gastaut syndrome; short-term evaluation.
Gaston et al. (2021) [81]	Open-label expanded-access program	169 patients (89 children and 80 adults) with DRE	Oral CBD, 5 mg/kg/day titrated up to 50 mg/kg/day for 2 years	Sustained reductions in seizure frequency (61% in children and 71% in adults) and seizure severity (75% in children and 85% in adults) during long-term treatment. Improvement observed in both pediatric and adult patients. CBD maintained a favorable safety profile throughout follow-up.	Open-label design; no placebo control; long-term observational study.
Morales et al. (2021) [82]	Observational EEG connectivity study	16 patients with DRE-encephalopathy (*n* = 6 receiving CBD; *n* = 10 without CBD) and matched healthy controls	Oral CBD, 25–50 mg/kg/day for 6–12 months	CBD treatment was associated with increased synchronization across EEG frequency bands and alterations in functional brain network organization; improved EEG organization and brain function. Findings suggest potential modulation of cortical connectivity associated with CBD therapy.	Very small sample size; non-randomized design; surrogate EEG outcomes rather than clinical efficacy.
McCoy et al. (2018) [86]	Open-label interventional trial	20 pediatric patients with DS and DRE; mean age ≈ 10 years	Cannabis extract containing 100 mg/mL CBD and 2 mg/mL THC; Oral CBD ~13.3 mg/kg/day for 20 weeks	Median motor seizure reduction of 70.6%. 63% of patients achieved ≥50% seizure reduction. Significant improvement in EEG interictal spike index and quality of life. Transient adverse events only.	Small sample size; open-label design; concomitant THC administration.

CBD, cannabidiol; DRE, drug-resistant epilepsy; DS, Dravet syndrome; EEG, electroencephalography; LGS, Lennox–Gastaut syndrome; RCT, randomized controlled trial; THC, Δ^9^-tetrahydrocannabinol.

Moreover, Contin et al. evaluated the pharmacokinetics of highly purified oral CBD in 43 patients (mean age, 26 ± 15 years) with drug-resistant DS and LGS enrolled in an expanded access program. Patients received a mean CBD dose of 13.2 ± 4.6 mg/kg/day. The median trough plasma concentration was 91 ng/mL, rising to 190 ng/mL approximately 2.5 h after administration. Plasma exposure demonstrated a linear correlation with the weight-adjusted daily dose. Age significantly influenced CBD pharmacokinetics, with adults exhibiting roughly 32% higher concentration-to-dose ratios than children, while gender and concomitant antiseizure medications had no significant effect. These findings indicate that older patients may require lower weight-adjusted doses to achieve comparable systemic exposure, supporting individualized dosing strategies in treatment-resistant epilepsy [87].

In epileptic encephalopathy, EEG-based functional connectivity analyses have demonstrated widespread increases in synchronization across most frequency bands, consistent with network hyperexcitability and large-scale abnormal integration, while connectivity in the alpha band appears relatively reduced compared with healthy controls. Adjunctive treatment with CBD was associated with measurable changes in these connectivity patterns, indicating a shift toward a more integrated and less segregated network organization. Such findings suggest that CBD may modulate pathological network dynamics and partially normalize aberrant functional connectivity [82]. Refractory or drug-resistant epilepsy (DRE) affects approximately one-third of pediatric patients and remains associated with substantial morbidity despite the availability of multiple antiseizure medications. This persistent therapeutic gap has prompted increasing interest in alternative and adjunctive treatment strategies, including cannabinoid-based pharmacotherapies. Among these, CBD has emerged as a particularly important candidate due to its broad anticonvulsant profile and lack of psychoactive effects. Preclinical and clinical evidence suggests that CBD exerts antiseizure activity through multiple complementary mechanisms. These include modulation of the endocannabinoid system, antagonism of excitatory receptor signalling, inhibition of intracellular calcium influx and downstream signalling cascades, and interactions with voltage-gated ion channels and diverse neurotransmitter systems [83,84]. Collectively, these effects are thought to reduce neuronal hyperexcitability, thereby contributing to seizure suppression. The multimodal pharmacodynamic profile of CBD distinguishes it from conventional antiseizure drugs and may contribute to its therapeutic utility in pharmacoresistant epilepsies. Among the neurological disorders reviewed, epilepsy is supported by the strongest body of clinical evidence, including multiple randomized controlled trials demonstrating the efficacy and safety of cannabidiol in patients with specific drug-resistant epilepsy syndromes.

## 6. Therapeutic Potential of CBD in the Management of Alzheimer’s Disease

Alzheimer’s disease (AD) is a progressive neurodegenerative disorder characterized by cognitive decline. Key pathological features include extracellular accumulation of β-amyloid (Aβ) plaques that disrupt synaptic communication and promote neuroinflammation, intracellular aggregates of hyperphosphorylated Tau forming neurofibrillary tangles that impair axonal transport, increased oxidative stress, chronic microglial activation, and extensive neuronal loss [88]. Oxidative stress is a central contributor to AD pathology, exacerbating Aβ accumulation, Tau hyperphosphorylation, and neuronal loss. In this context, Kim et al. [89] investigated the effects of CBD on primary hippocampal neurons derived from embryonic day 18 Sprague–Dawley rats. Neurons were exposed to CBD (0.1–100 μM) and/or hydrogen peroxide (H_2_O_2_, 0.1–50 μM) for 24 h, with median lethal concentration (LC_50_) values of 9.85 μM and 2.46 μM, respectively, indicating the greater inherent toxicity of oxidative stress induced by H_2_O_2_ compared with CBD. Notably, CBD exhibited a biphasic response: low concentrations (≤5 μM) enhanced neuronal survival under oxidative stress, whereas higher concentrations (>5 μM) induced cytotoxicity. The neuroprotective effects are hypothesized to result from CBD’s antioxidant properties and its ability to maintain cell membrane integrity, although the precise mechanisms remain to be fully elucidated. These findings suggest that CBD may attenuate oxidative neuronal damage, a key pathogenic mechanism in AD, while underscoring the importance of careful dose selection for therapeutic application [89].

The main preclinical studies investigating the therapeutic potential of CBD in Alzheimer’s disease are summarized in Table 3.

Esposito et al. provided one of the first in vivo demonstrations of the anti-inflammatory properties of CBD in a model relevant to AD. Following intrahippocampal injection of β-amyloid (Aβ_1–42_) to induce localized neuroinflammation and reactive gliosis in mice, intraperitoneal administration of CBD (2.5–10 mg/kg, i.p.) attenuated astrocyte activation, as evidenced by reduced glial fibrillary acidic protein (GFAP) expression. CBD also suppressed the expression of key pro-inflammatory mediators, including inducible nitric oxide synthase (iNOS) and interleukin-1β (IL-1β), accompanied by decreased nitric oxide (NO) production and cytokine release in hippocampal tissue. These findings indicate that CBD attenuates Aβ-induced glial activation and inflammatory signalling in this experimental model, supporting the hypothesis that modulation of neuroinflammation contributes to its neuroprotective profile and suggesting its potential therapeutic relevance for Alzheimer’s disease-associated pathology [90]. Extending their previous studies, the same authors provided further evidence that CBD attenuates Aβ-induced neuroinflammation and enhances hippocampal neurogenesis. In primary rat astrocyte cultures exposed to Aβ (1 µg/mL), CBD (10^−9^–10^−7^ M) reduced the levels of pro-inflammatory mediators (NO, IL-1β, tumor necrosis factor α—TNFα), iNOS and GFAP expression, and nuclear factor NF-κB activation. These effects were reversed by the PPARγ antagonist GW9662 but not by a peroxisome proliferator-activated receptor alpha (PPARα) antagonist. In the corresponding animal model, adult rats receiving bilateral intrahippocampal Aβ injections and daily i.p. CBD (10 mg/kg/day, for 15 days) showed significantly reduced Aβ-induced gliosis and neuronal loss together with increased dentate gyrus neurogenesis. Co-treatment with a PPARγ antagonist (10 mg/kg, i.p.) abolished both the anti-inflammatory and neurogenic actions of CBD. These findings suggest that PPARγ activation may be a key mediator of CBD’s anti-inflammatory and neuroprotective actions in Aβ-related pathology [91]. Other preclinical studies also indicate that CBD attenuates microglial activation and neuroinflammation associated with AD. In vitro, CBD suppresses proinflammatory signalling in microglia, while in rodent models of β-amyloid pathology, it preserves cognitive function and reduces the expression of inflammatory cytokine genes [92]. These findings suggest the multimodal neuroprotective properties of CBD and support its potential as a non-psychoactive therapeutic approach in AD.

Cheng et al. reported that chronic CBD administration (8 months) attenuated the development of social recognition memory deficits in AβPP × PS1 transgenic mice. Long-term CBD treatment prevented the emergence of this specific cognitive impairment without significantly affecting other behavioural domains, including anxiety-like behaviour and associative learning. This selective improvement suggests domain-specific neuroprotective effects rather than broad cognitive enhancement. CBD exposure was also associated with subtle alterations in brain cholesterol and phytosterol profiles, suggesting a possible link between sterol homeostasis and CBD-mediated cognitive protection [93]. Disruption of cerebral cholesterol metabolism is increasingly recognized as a key contributor to AD pathogenesis. Cholesterol is essential for neuronal membrane integrity, synaptic function, and lipid raft organization, all of which influence amyloid precursor protein (APP) processing and Aβ generation. While physiological cholesterol homeostasis supports neuronal resilience, imbalances in cholesterol turnover or distribution have been linked to enhanced amyloidogenic processing, synaptic dysfunction, and neurodegeneration. Consequently, interventions that stabilize brain sterol metabolism may provide neuroprotective benefits [94]. Phytosterols, plant-derived sterols structurally similar to cholesterol, constitute an additional class of molecules that may influence brain lipid biology. Although their ability to penetrate across the blood–brain barrier appears limited, measurable levels have been detected in the brain, where they may interact with cholesterol metabolic pathways. Observational and experimental studies suggest that higher circulating concentrations of certain phytosterols, particularly sitosterol and stigmasterol, are associated with favourable lipid profiles, reduced systemic inflammation and oxidative stress, and, in some cohorts, better cognitive performance or a lower AD risk [95]. Preclinical studies further indicate that phytosterols may modulate APP processing by potentially reducing β- and γ-secretase activity and decreasing Aβ production. Their reported anti-inflammatory and antioxidant properties may also contribute to their neuroprotective effects [96]. Taken together, these findings suggest that circulating and brain sterols may represent both potential biomarkers and therapeutic targets in AD, although causal relationships remain to be established in humans [95]. In this context, the observation that CBD subtly alters brain sterol composition raises the possibility that sterol modulation may partly mediate its protective effects on social memory. This hypothesis highlights lipid homeostasis as an underexplored mechanistic pathway that complements classical therapeutic targets centred on amyloid pathology and oxidative stress. Greater integration of lipid biology into AD research may therefore improve our understanding of disease mechanisms and promote the development of multifaceted therapeutic strategies [93].

In a transgenic *Caenorhabditis elegans* Aβ42 model (strain CL4176), Zhang et al. found that CBD (0–100 µM in culture medium) mitigates Aβ-associated toxicity in a concentration-dependent manner, delaying paralysis onset and reducing Aβ aggregation in vivo. Importantly, CBD did not inhibit β-sheet formation or fibrillogenesis in vitro, arguing against a direct anti-amyloid mechanism. Instead, protection was attributed to its intrinsic antioxidant activity: CBD lowered intracellular reactive oxygen species (ROS) and conferred resistance to oxidative stress without upregulating classical antioxidant gene networks such as catalases, superoxide dismutases, or glutathione S-transferases, or requiring major stress-responsive transcription factors such as DAF-16 and SKN-1. Mechanistic analysis revealed that the phenolic hydroxyl groups of CBD are essential for its ROS-scavenging capacity and related neuroprotective effects, highlighting the importance of its intrinsic antioxidant properties rather than modulation of endogenous defence pathways [97]. These findings provide mechanistic insight into the mode of action of CBD and indicate that its chemical structure, especially its phenolic functional groups, may play a critical role in the development of antioxidant-based therapeutic strategies for AD.

In a more comprehensive preclinical evaluation, Raich et al. demonstrated that CBD exerts multifaceted neuroprotective effects across several key mechanisms implicated in AD pathology. In primary neuronal cultures, CBD (200 nM) significantly reduced the aggregation of Aβ, Tau, and phosphorylated Tau (pTau) proteins by 61%, 82% and 69%, respectively. These reductions were accompanied by decreased reactive oxygen species (ROS) production and improved neuronal viability under toxic conditions. Neurons exposed to Aβ (500 nM) and N-methyl-D-aspartate (NMDA, 30 µM) exhibited about 45% and 40% cell death, respectively, which was substantially counteracted after 48 h of treatment with CBD (200 nM), indicating a marked improvement in neuronal survival. Furthermore, CBD limited the axonal transport of Aβ, Tau and pTau between cortical and hippocampal neurons, suggesting a potential mechanism for restricting pathological spread. In the animal model, systemic CBD administration (i.p., 10 mg/kg/day for 28 days) in 5xFAD transgenic mice ameliorated both neuropathological and cognitive deficits. Therefore, CBD treatment reduced cortical and hippocampal Aβ plaque burden; modulated neuroinflammation by shifting microglia toward a neuroprotective M2-like phenotype; decreased the release of pro-inflammatory cytokines (e.g., IL-1β); reduced astroglial activation, decreased GFAP expression and increased the number of oligodendrocyte precursor cells; and improved both short-term and long-term spatial memory in the novel object recognition test (NORT). Consistent effects were observed in the CL2006 *C. elegans* AD model, where CBD (10 µM and 100 µM) increased locomotor performance, reduced Aβ deposition, and delayed paralysis onset. These findings indicate that CBD targets multiple pathological processes, including protein aggregation, oxidative stress, neuroinflammation, neuronal loss, and cognitive impairment, suggesting its potential as a multitarget therapeutic candidate for AD. Receptor-blocking experiments showed that CBD’s neuroprotective effects are largely CB1-dependent in neurons, whereas its anti-inflammatory and microglial-modulating actions require both CB1 and CB2 receptors, with a stronger contribution from CB2R [98].

In a recent preclinical study, Toledano and Akirav evaluated the preventive effects of chronic CBD administration in the intracerebroventricular streptozotocin (ICV-STZ) rat model of sporadic AD. Unlike most previous studies employing higher or therapeutic dosing paradigms, CBD was administered at a low dose (0.1 mg/kg/day, i.p.) for 14 days starting immediately after STZ injection. STZ produced marked behavioural impairments, including deficits in recognition and spatial memory, evidenced by significant reductions in novel object recognition and object location discrimination indices (~40–50% relative to controls), as well as decreased sociability (~30–40%). These behavioural deficits were accompanied by pronounced neuropathological alterations in the hippocampus, including increased levels of Aβ and p-Tau (~50–80% elevations), as well as an upregulation of proinflammatory mediators such as TNF-α, NF-κB1, and IL-1β (~1.5–2.5-fold increases). Chronic CBD treatment largely prevented these deficits. Therefore, CBD-treated animals displayed almost complete restoration of cognitive and social performance, with discrimination scores returning to ~90–100% of control values. At the molecular level, CBD attenuated AD-like pathology, reducing Aβ and p-Tau accumulation by ~30–50% compared with STZ-treated rats and normalizing the expression of inflammatory mediators to baseline. Importantly, locomotor activity and anxiety-like behaviours were not affected, indicating that the observed cognitive improvements were not secondary to changes in general behavioural. STZ also disrupted hippocampal endocannabinoid signalling by altering cannabinoid receptor expression, whereas CBD restored both CB1 and CB2 receptor levels. Pharmacological interrogation demonstrated receptor specificity: co-administration of a CB1 antagonist (e.g., AM251) abolished the cognitive and social benefits of CBD, while the co-administration of CB2 antagonists (e.g., AM630) had no effect. These findings indicate that the neuroprotective and anti-inflammatory actions of CBD in this model are primarily mediated by a CB1-dependent mechanism. Consequently, short-term treatment with low doses of CBD produced significant preventive effects against both behavioural deficits and molecular hallmarks of AD-like pathology, suggesting that modulation of the endocannabinoid system, particularly CB1 signalling, may represent an early therapeutic strategy for neurodegenerative disorders [99].

**Table 3:** Summary of preclinical studies investigating the effects of CBD in Alzheimer’s disease models.

Study	Study Design	Experimental Model	CBD Regimen	Main Findings	Limitations
Kim et al. (2021) [89]	In vitro oxidative stress model	Primary hippocampal neurons from embryonic day-18 Sprague–Dawley rats	CBD 0.1–100 μM for 24 h; H_2_O_2_-induced oxidative stress	Low CBD concentrations (≤5 μM) improved neuronal viability under H_2_O_2_-induced oxidative stress (57% viability with CBD vs. 24% with H_2_O_2_ alone). High CBD concentrations (>5 μM) were cytotoxic. Demonstrated dose-dependent neuroprotective and antioxidant effects.	In vitro model; short exposure; limited translational relevance.
Esposito et al. (2007) [90]	In vivo experimental study	C57BL/6J mice with right intrahippocampal Aβ(1–42) injection	CBD 2.5 or 10 mg/kg/day (i.p.) for 7 days	Reduced GFAP, iNOS, IL-1β and NO production. Attenuated Aβ-induced neuroinflammation and oxidative stress.	Acute animal model; short treatment duration.
Esposito et al. (2011) [91]	In vivo experimental study	Adult Sprague–Dawley rats with bilateral intrahippocampal Aβ(1–42) injection	CBD 10 mg/kg/day (i.p.) for 15 days	Reduced neuroinflammation and neuronal damage. Enhanced hippocampal neurogenesis. Effects mediated through PPARγ activation.	Animal model; mechanistic findings require clinical confirmation.
Martin-Moreno et al. (2011) [92]	In vivo experimental study	C57BL/6 mice with intracerebroventricular Aβ(1–40) injection	CBD 20 mg/kg/day (i.p.) for 7 days, followed by 20 mg/kg three times/week for 2 weeks	Prevented Aβ-induced cognitive impairment. Reduced IL-6 expression. Preserved cognitive function by attenuating neuroinflammation.	Preclinical study; relatively short follow-up.
Cheng et al. (2014) [93]	In vivo experimental study	APP×PS1 transgenic mice	CBD 20 mg/kg/day orally for 8 months	Prevented social recognition memory deficits. Increased cortical cholesterol and phytosterols. No significant effects on anxiety, amyloid load or inflammatory cytokines.	Limited behavioral benefit; no reduction in amyloid burden.
Zhang et al. (2022) [97]	In vivo experimental study	*Caenorhabditis elegans* Aβ42 model (CL4176)	CBD 0–100 μM in nematode growth medium	Reduced Aβ-induced toxicity and delayed paralysis. Decreased ROS and improved resistance to oxidative stress. Inhibited Aβ aggregation, visible after 7 days.	Non-mammalian model; uncertain clinical translation.
Raich et al. (2025) [98]	In vitro and in vivo experimental study	Primary neurons from 5×FAD transgenic mice 5×FAD mice *C. elegans* AD model (CL2006)	CBD 200 nM (in vitro) 10 mg/kg/day (i.p.) for 28 days (in vivo) 10–100 μM in nematode growth medium (in vivo)	Reduced axonal spread of Aβ, Tau and pTau. Shifted microglia toward an anti-inflammatory M2 phenotype. Reduced neuroinflammation (decreased IL-1β and GFAP levels), improved cognitive performance. Delayed paralysis onset, increased locomotor performance and reduced Aβ deposition in *C. elegans*.	Preclinical evidence only; multiple experimental models increase complexity of interpretation.
Toledano et al. (2025) [99]	In vivo experimental study	ICV-STZ rat model of sporadic AD	CBD 0.1 mg/kg/day (i.p.) for 14 days	Prevented cognitive and social deficits (~90–100% vs control). Reduced hippocampal Aβ and pTau (~30–50% vs control) and neuroinflammatory markers (TNF-α, IL-1β, NF-κB). Effects primarily mediated through CB1 receptor activation.	Animal model; preventive rather than therapeutic treatment design.

Aβ, amyloid-beta; AD, Alzheimer’s disease; CBD, cannabidiol; GFAP, glial fibrillary acidic protein; H_2_O_2_, hydrogen peroxide; i.p., intraperitoneal; iNOS, inducible nitric oxide synthase; IL-1β, interleukin-1 beta; NF-κB, Nuclear factor kappa B; NO, nitric oxide; PPARγ, peroxisome proliferator-activated receptor gamma; pTau, phosphorylated Tau; ROS, reactive oxygen species; TNF-α, Tumor necrosis factor alpha.

AD is a progressive neurodegenerative disorder with serious consequences for the patient. Current pharmacological treatments, including acetylcholinesterase inhibitors and memantine, an NMDA receptor antagonist, provide only modest symptomatic relief and do not halt disease progression. Preclinical studies indicate that CBD exerts anti-inflammatory, antioxidant, and neuroprotective effects in cellular and animal models of AD. In vitro and in vivo experiments show that CBD reduces markers of neuroinflammation (e.g., IL-1β, TNF-α, iNOS, and NF-κB) and attenuates glial activation, reflected by decreased GFAP expression. CBD also limits oxidative stress by reducing intracellular ROS and lipid peroxidation and increasing resistance to oxidative stress. In addition, CBD may influence amyloidogenic processing by decreasing APP and BACE1 (β-site Amyloid Precursor Protein Cleaving Enzyme) expression and limiting the pathological propagation of Tau and phosphorylated Tau between neurons, collectively targeting key pathogenic pathways in Alzheimer’s disease. Mechanistically, these benefits have been linked to the modulation of endocannabinoid tone, signalling mediated by CB1/CB2 receptors as well as activation of non-cannabinoid receptors such as PPARγ. Emerging evidence further suggests that disturbances in brain lipid and sterol metabolism may contribute to early synaptic dysfunction and amyloid pathology, and some animal studies indicate that CBD may subtly influence sterol homeostasis, which may contribute to its neuroprotective profile. The available preclinical evidence suggests that CBD may attenuate neuronal damage and help preserve cognitive function, although confirmation in well-designed clinical studies is still required. Evidence supporting the use of CBD in Alzheimer’s disease remains largely preclinical, while clinical data are still insufficient to establish therapeutic efficacy.

## 7. Therapeutic Potential of CBD in Parkinson’s Disease

Parkinson’s disease (PD) is a chronic, progressive neurodegenerative disorder marked by dopaminergic neuronal loss in the *substantia nigra pars compacta* (SNc). This dopamine depletion disrupts motor control and leads to hallmark symptoms such as resting tremor, muscle rigidity, bradykinesia, postural instability, and coordination difficulties. Non-motor symptoms are also common and may include anxiety, cognitive impairment, sleep disturbances, and, particularly in later stages or during pharmacological treatment, psychosis [100].

The main clinical and preclinical studies investigating the therapeutic potential of CBD in Parkinson’s disease are summarized in Table 4.

In 2011–2012, Lotan et al. conducted a small study to explore the acute effects of inhaled medicinal cannabis on motor and non-motor symptoms in 22 patients with PD. Clinical assessments were performed immediately before and 30 min after smoking cannabis. Motor function, as measured by the motor subsection of the Unified Parkinson’s Disease Rating Scale (UPDRS), improved significantly (≈30% improvement). Significant reductions in tremor, rigidity, and bradykinesia were observed. Improvements in non-motor symptoms, such as pain intensity, sleep quality, and overall well-being, were also reported, with no serious adverse events. These preliminary results suggest a rapid symptomatic benefit of cannabis, through its constituent compounds, for both motor and selected non-motor manifestations of PD [101]. In addition, in an anonymous web-based survey, Kindred et al. examined the prevalence, patterns, and self-reported effects of cannabis use among people with PD and multiple sclerosis (MS). A total of 595 respondents (≈76% PD; ≈24% MS) completed standardized questionnaires assessing disability, fatigue, mood, memory, balance confidence, and physical activity. Current cannabis use was reported by approximately 44% of participants. Cannabis users reported high perceived efficacy (mean 6.4 ± 1.8 on a scale of 0 to 7), and 59% indicated reduced use of prescription medications after initiating cannabis use. Relative to non-users, users demonstrated lower self-reported disability, particularly in fatigue, mood, and memory, with no significant differences observed for balance confidence or physical activity [102]. Because outcomes were self-reported, the findings indicate associations rather than therapeutic effects; however, the frequency of use and consistent self-reported symptomatic improvement suggest that cannabis use is common as an adjunctive therapy in PD and MS and support the need for prospective, controlled trials to determine its efficacy and safety.

Further in vitro studies showed that CBD exerts quantitatively measurable neuroprotective effects against dopaminergic toxicity induced by various substances, such as the toxin MPP^+^ (1-methyl-4-phenylpyridium), known to induce parkinsonism in vivo. While MPP^+^ exposure reduced PC12 cell viability by approximately 50–60%, CBD (≈1–10 µM) significantly attenuated this loss, increasing survival by ~10–20% compared with the MPP^+^ control group and bringing viability close to baseline values at optimal doses. CBD also promoted neuronal differentiation, increasing the proportion of neurite-bearing cells by 30–50% and significantly enhancing neurite length and branching compared with MPP^+^ alone. At the molecular level, CBD upregulated axonal and synaptic markers, including growth-associated protein GAP-43 and the presynaptic proteins synaptophysin and synapsin I, with reported increases of approximately 1.5–2.5-fold, suggesting structural repair and functional support of neurons. Pharmacological blockade of TrkA (tropomyosin receptor kinase A) receptors significantly reduced these protective and neuritogenic effects, suggesting that CBD’s actions depend on neurotrophin-like signalling rather than exclusively on antioxidant mechanisms. These data support CBD as a modulator of both neuronal survival and structural plasticity in cellular models relevant to PD neurodegeneration [103].

Giuliano et al. investigated the neuroprotective and symptomatic effects of chronic CBD administration in a unilateral 6-hydroxydopamine (6-OHDA)-induced rat model of PD. In placebo-treated animals, the lesion induced severe degeneration of the nigrostriatal pathway. CBD significantly attenuated this pathology, reducing both striatal dopaminergic terminal loss and neuronal loss in the substantia nigra by 21% relative to the control group, indicating a measurable neuroprotective effect on nigrostriatal dopaminergic neurons. Behavioural assessments revealed parallel functional benefits: CBD reduced forelimb asymmetry in the cylinder test by 34% and decreased apomorphine-induced contralateral rotations by 54%, suggesting partial restoration of motor function in lesioned animals. Inflammatory profiling revealed minimal effects on microglial density or phenotype; however, a significant 14% reduction in GFAP^+^ astrocyte density was observed in the lesioned substantia nigra, indicating attenuation of astrocytic reactivity. Molecular analyses further indicated increased astrocytic expression of transient receptor potential vanilloid 1 (TRPV1) receptors and elevated levels of ciliary neurotrophic factor (CNTF) following CBD treatment, suggesting the partial involvement of astrocyte-mediated neurotrophic signalling [104]. These results indicate that chronic CBD administration confers measurable neuroprotection to dopaminergic neurons and produces selective motor improvements in this progressive experimental model of PD. The observed benefits appear to be associated with reduced astrocyte reactivity and enhanced astrocytic TRPV1/CNTF signalling, suggesting that CBD may exert both disease-modifying and symptomatic effects in parkinsonian neurodegeneration.

In clinical trials, the effects of CBD have been investigated at various concentrations. An open-label pilot study in PD patients showed that oral CBD doses of 150–400 mg/day, when added to standard antiparkinsonian therapy, were associated with a reduction in psychotic symptoms as measured by scales such as the Brief Psychiatric Rating Scale (BPRS) and the Parkinson’s Psychosis Questionnaire (PPQ), with no significant impact on motor or cognitive function and no serious adverse effects. Although limited by the small sample size (*n* = 6) and open-label design, these preliminary findings indicate an improvement in hallucinations and delusions associated with PD psychosis [105]. The study also suggests a potential antipsychotic effect of CBD in PD, without exacerbating motor impairment. This is clinically important because many antipsychotics worsen parkinsonian motor symptoms. When CBD was tested in patients with PD at doses of 75 mg/day and 300 mg/day, respectively, no group differences were found in motor function or overall disease severity (UPDRS), or in biomarkers, including plasma levels of brain-derived neurotrophic factor (BDNF) and proton magnetic resonance spectroscopy (MRS) measurements. However, patients receiving 300 mg/day showed improved quality of life, reflected by a reduction in total scores on the Parkinson’s disease Questionnaire-39 (PDQ-39) compared with placebo. Treatment with 300 mg/day of CBD also improved mobility, emotional well-being, communication, and body discomfort compared to placebo, suggesting benefits in multiple domains, potentially related to CBD’s anxiolytic, antidepressant, and antipsychotic properties [106]. Additionally, in studies conducted with the same doses, CBD reduced the frequency of REM sleep behaviour disorder events, a distressing non-motor symptom of PD [107]. Furthermore, low-dose CBD administration (26 mg/day) did not result in statistically significant improvements in global cognitive function, motor outcomes, neuropsychiatric measures, or circulating inflammatory markers in patients with PD. Although a statistically significant increase in the naming subdomain of the Montreal Cognitive Assessment (MoCA) was observed in the CBD group compared with placebo, this isolated effect should be interpreted with caution, especially in the absence of consistent benefits in other cognitive domains. Overall, these results indicate that low doses of CBD have a limited impact in this context, although it may exert selective effects on certain cognitive functions [108]. Taken together with previous studies, these findings suggest that higher-dose CBD may improve patient-reported quality of life without affecting motor symptoms or biological markers, highlighting the need for larger, adequately powered clinical trials to confirm these preliminary observations [106].

However, CBD has recently been investigated as a potential therapeutic option for the motor and non-motor symptoms of PD. In an open-label study, Leehey et al. evaluated the safety, tolerability, and preliminary efficacy of purified CBD in patients with PD and resting tremor. CBD (Epidiolex) was administered in escalating doses from 5 mg/kg/day to a maximum dose of 20–25 mg/kg/day over several days. Although the participants reported adverse events such as diarrhoea, somnolence, and fatigue, there were generally no serious adverse events or withdrawal symptoms. Significant improvements in motor outcomes were observed in the total UPDRS score (17.8%) and in the motor subscale (24.7%). Improvements in nocturnal sleep parameters were also reported. Although limited by the small sample size and open-label design, the study suggests that high doses of CBD may provide modest improvements in motor and sleep symptoms in PD, while emphasizing the need for careful monitoring of gastrointestinal and hepatic adverse effects [109]. Continuing this line of investigation, the authors conducted double-blind, placebo-controlled clinical trials (Clinical Trial NCT0358213—61 patients [110] and Clinical Trial NCT03582137—58 patients [111]) to evaluate the short-term (about 2 weeks) effects of an oral cannabinoid formulation containing high-dose CBD (100 mg/mL) and low-dose THC (3.3 mg/mL) administered up to twice daily. The target dose was 2.5 mg/kg/day, corresponding to a mean achieved dose of 191.8 ± 48.9 mg/day of CBD and 6.4 ± 1.6 mg/day of THC. The primary outcome, the change in Movement Disorder Society Unified Parkinson’s disease Rating Scale (MDS-UPDRS) Part III motor score, improved significantly from baseline in both the active treatment group (−4.57) and the placebo group (−2.77); however, the between-group difference was not statistically significant, indicating no superiority of the cannabinoid intervention over placebo [110]. Secondary outcomes assessing sleep, cognition, and activities of daily living showed no benefit and, in some cases, favoured placebo, suggesting potential negative effects on non-motor symptoms. Adverse events were more frequent in the treatment group, although predominantly mild, with possible worsening of cognition and sleep [110,111]. These findings indicate that short-term exposure to a combined CBD/THC regimen did not provide clinically meaningful improvement in motor symptoms in PD and did not demonstrate cognitive benefit, supporting a cautious interpretation of these findings and the use of cannabinoid-based therapy in this population.

**Table 4:** Summary of clinical and preclinical studies investigating the therapeutic potential of CBD in Parkinson’s disease.

Study	Study Design	Subjects/ Experimental Model	CBD Regimen	Main Findings	Limitations
Lotan et al. (2014) [101]	Open-label observational clinical study	22 patients with PD; 65 ± 10.2 years old	Smoked cannabis (0.5 g); treatment for at least 2 months	Improved UPDRS score and major motor symptoms (tremor, rigidity, bradykinesia). Improved sleep quality and pain. No significant adverse effects reported.	Open-label design; no placebo control; small sample size; cannabis rather than purified CBD.
Santos et al. (2015) [103]	In vitro experimental study	PC12 cells exposed to MPP^+^	CBD 1–10 μM	Increased cell viability and neuronal survival. Reduced caspase-3 activity. Preserved neurite outgrowth and increased synaptic protein expression. Demonstrated neurotrophic and neuroprotective effects via Trk-dependent mechanisms.	In vitro model; uncertain translation to clinical disease.
Giuliano et al. (2021) [104]	In vivo experimental study	6-OHDA rat model of PD	CBD 10 mg/kg/day (i.p.) for 28 days	Reduced dopaminergic neuron loss (21% vs. 70% in controls). Improved selected motor outcomes. Reduced astrocyte activation. Activated TRPV1-CNTF neuroprotective signaling.	Animal model; limited improvement in some motor tests (e.g., rotarod performance).
Zuardi et al. (2009) [105]	Open-label pilot clinical study	6 patients with PD and psychosis; mean age 58.8 ± 14.9 years	Oral CBD 150–400 mg/day for 4 weeks	Marked reduction in psychotic symptoms (BPRS, PPQ). No worsening and possible slight improvement of motor symptoms. CBD was well tolerated.	Very small sample size; no placebo control.
Chagas et al. (2014) [106]	Double-blind, placebo-controlled exploratory trial	21 patients with PD; ˃45 years old	Oral CBD 75 mg (*n* = 7) or 300 mg/day (*n* = 7) for 6 weeks	No significant improvement in motor symptoms and neuroprotection markers. Improved quality of life (PDQ-39) with 300 mg/day. No major safety concerns.	Small exploratory trial; short treatment duration.
Mitarnun et al. (2025) [108]	Randomized, double-blind, placebo-controlled trial	60 patients with PD (51 patients completed the study)	Sublingual CBD-enriched extract (~26 mg/day CBD, trace THC ~1.2 mg/day) for 12 weeks	Improved naming ability on MoCA. No significant effects on motor symptoms, mood or inflammatory markers. Favorable safety profile; no detectable THC in plasma.	Moderate sample size; cognitive improvements limited to selected domains.
Leehey et al. (2020) [109]	Open-label dose-escalation clinical study	13 patients with PD (10 patients completed the study); mean age 68.1 ± 6.1 years	Oral CBD (Epidiolex, 100 mg/mL) 5 mg/kg/day escalating to 20–25 mg/kg/day; highest tolerated dose maintained for 10–15 days.	Significant improvements in total and motor UPDRS scores (17.8% and 24.7%, respectively). Improved nighttime sleep and behavioral symptoms. Adverse events were mostly mild; no serious adverse events occurred.	Open-label design; high incidence of mild adverse effects; small cohort.

6-OHDA, 6-hydroxydopamine; BPRS, Brief Psychiatric Rating Scale; CBD, cannabidiol; CNTF, Ciliary Neurotrophic Factor; i.p., intraperitoneal; MoCA, Montreal Cognitive Assessment; MPP^+^, 1-methyl-4-phenylpyridinium; PD, Parkinson’s disease; PDQ-39, Parkinson’s Disease Questionnaire-39; PPQ, Parkinson Psychosis Questionnaire; Trk, tropomyosin receptor kinase; TRPV1, transient receptor potential vanilloid 1; THC, Δ^9^-tetrahydrocannabinol; UPDRS, Unified Parkinson’s Disease Rating Scale.

Overall, current clinical evidence suggests that CBD is generally well tolerated in patients with Parkinson’s disease and may have beneficial effects on non-motor symptoms such as psychosis, anxiety, sleep disturbances, emotional well-being, and discomfort. While moderate doses have been associated with improvements in quality of life, high doses of CBD have demonstrated acceptable tolerability, accompanied by preliminary evidence of improvement in motor symptoms. Therefore, cannabinoids may represent a potential adjunctive therapy for the management of both motor and non-motor symptoms in Parkinson’s disease. However, evidence for clinically meaningful improvements in core motor symptoms remains limited and inconsistent, highlighting the need for larger, well-controlled randomized trials to confirm efficacy, optimal dosing, and long-term safety. Clinical evidence is currently limited to a small number of pilot studies and randomized clinical trials with modest sample sizes, preventing firm conclusions regarding therapeutic efficacy.

## 8. Methodology

This review was conducted in accordance with the Preferred Reporting Items for Systematic Reviews and Meta-Analyses (PRISMA 2020) guidelines. The literature search was performed using ISI Web of Science, PubMed, ScienceDirect, and Scopus databases and included articles published between January 2000 and November 2025. The search strategy combined Medical Subject Headings (MeSH), where applicable, and free-text terms, including *Cannabis sativa*, cannabidiol, anxiety, mood disorders, sleep disorders, epilepsy, Alzheimer’s disease, Parkinson’s disease, COVID-19, endocannabinoid system, and cannabinoids. Additional relevant publications were identified through manual screening of the reference lists of eligible articles.

**Figure 2:**
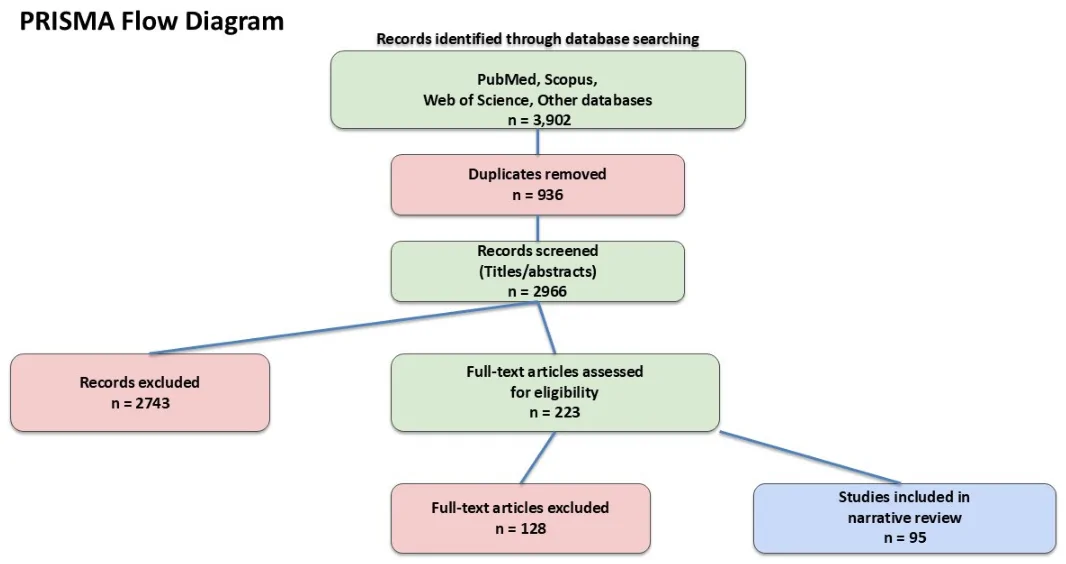
PRISMA 2020 flow diagram illustrating the literature search and study selection process.

Original research articles, clinical trials, observational studies, and relevant preclinical investigations were considered for inclusion. Editorials, conference abstracts, duplicate publications, and studies without sufficient methodological information were excluded. Titles, abstracts, and subsequently full-text articles were screened for eligibility according to predefined inclusion and exclusion criteria. The study selection process is summarized in the PRISMA 2020 flow diagram (Figure 2).

Although this review followed the PRISMA guidelines for study identification and selection, no formal risk-of-bias assessment was performed because the objective was to provide a broad overview of the available evidence across several heterogeneous mental and neurological disorders rather than to conduct a quantitative evidence synthesis. Consequently, the findings were interpreted in the context of study design, sample size, methodological limitations, and consistency of the reported results.

## 9. Conclusions

Plant biodiversity represents a valuable source of bioactive compounds and continues to provide opportunities for the development of novel therapeutic strategies.

Phytocannabinoids are lipophilic compounds that are rapidly absorbed by the body. Most available pharmacokinetic data pertain to CBD and THC. The pharmacokinetic profiles of these compounds can vary considerably among individuals and are influenced by factors such as dosage, formulation, frequency of use (acute versus chronic), and route of administration. Inhalation methods, including smoking and vaporizing cannabis, result in higher blood concentrations of cannabinoids, a faster onset of action, and greater bioavailability than oral administration. Due to their high lipophilicity, natural cannabinoids exhibit strong membrane permeability and interact efficiently with the lipid-rich environment of CB1 and CB2 receptors. The lipophilic nature of these compounds is a key determinant of their pharmacokinetic properties, tissue distribution, and affinity for intracellular and mitochondrial receptor sites.

Among phytocannabinoids, CBD has received the greatest scientific attention because of its broad pharmacological profile and favourable safety characteristics. The available preclinical and clinical findings suggest that CBD may provide therapeutic benefits in epilepsy, anxiety disorders, and neurodegenerative diseases such as Alzheimer’s disease and Parkinson’s disease, although the strength of support varies considerably among these indications. Drug-resistant epilepsy is supported by the strongest clinical evidence, including multiple randomized controlled trials that led to the approval of CBD-containing medications such as Epidiolex, which has demonstrated significant reductions in seizure frequency and severity. In contrast, evidence for anxiety disorders, Alzheimer’s disease, Parkinson’s disease, and sleep disorders remains limited and is derived primarily from preclinical studies and relatively small clinical trials with heterogeneous study populations, treatment protocols, and outcome measures. These methodological differences limit direct comparisons among studies and make it difficult to establish optimal dosing regimens or accurately estimate treatment effects.

Across the disorders reviewed, CBD appears to act through multiple mechanisms, including modulation of the endocannabinoid system, serotonergic signaling, neuroinflammatory pathways, oxidative stress, and glutamatergic neurotransmission. These pleiotropic actions may explain its broad therapeutic potential, although the relative contribution of each mechanism is likely to vary according to the underlying disease pathophysiology.

The main limitation of the current evidence is its uneven quality across therapeutic indications. While epilepsy is supported by robust randomized clinical trials, studies investigating other mental and neurological disorders are generally characterized by small sample sizes, short follow-up periods, heterogeneous methodologies, and limited independent replication. Consequently, important questions remain regarding optimal dosing, treatment duration, long-term safety, drug–drug interactions, and the identification of patients most likely to benefit from CBD therapy.

CBD is a promising therapeutic candidate for several mental and neurological disorders. Nevertheless, its clinical use beyond approved indications requires confirmation through well-designed, multicenter randomized controlled trials and mechanistic studies capable of translating encouraging experimental findings into evidence-based clinical practice.

## Data Availability

No new data were created or analysed in this study. Data sharing is not applicable to this article.

## Abbreviations

The following abbreviations are used in this manuscript:

2-AG	2-Arachidonoylglycerol
5-HT1A	5-Hydroxytryptamine 1A receptor
Aβ	Amyloid-beta
AD	Alzheimer’s disease
AEA	Anandamide
AM251	Cannabinoid CB1 receptor antagonist
AM630	Cannabinoid CB2 receptor antagonist
APP	Amyloid precursor protein
BACE1	β-Site amyloid precursor protein-cleaving enzyme 1
BDNF	Brain-derived neurotrophic factor
BONSAI	Burnout and Distress Study (clinical trial acronym, if retained)
BPRS	Brief Psychiatric Rating Scale
CB1	Cannabinoid receptor type 1
CB2	Cannabinoid receptor type 2
CBD	Cannabidiol
CL2006	*Caenorhabditis elegans* AD model strain
CL4176	*Caenorhabditis elegans* AD model strain
CNS	Central nervous system
CNTF	Ciliary neurotrophic factor
COVID-19	Coronavirus disease 2019
DAF-16	Dauer formation protein 16
DALY	Disability-adjusted life year
DNA	Deoxyribonucleic acid
DNMT	DNA methyltransferase
DNMT1	DNA methyltransferase 1
DRE	Drug-resistant epilepsy
DS	Dravet syndrome
ECS	Endocannabinoid system
EEG	Electroencephalogram
EMA	European Medicines Agency
EPM	Elevated plus maze
FDA	U.S. Food and Drug Administration
FNE	Fear of Negative Evaluation
FST	Forced swim test
GAP-43	Growth-associated protein 43
GFAP	Glial fibrillary acidic protein
GPR18	G protein-coupled receptor 18
GPR55	G protein-coupled receptor 55
GW9662	PPARγ antagonist
H_2_O_2_	Hydrogen peroxide
ICV-STZ	Intracerebroventricular streptozotocin
IL-1β	Interleukin-1 beta
ILAE	International League Against Epilepsy
iNOS	Inducible nitric oxide synthase
LC50	Median lethal concentration
LGS	Lennox–Gastaut syndrome
LSAS	Liebowitz Social Anxiety Scale
MDS-UPDRS	Movement Disorder Society Unified Parkinson’s Disease Rating Scale
MPP^+^	1-Methyl-4-phenylpyridinium
MRS	Magnetic resonance spectroscopy *(verify against the cited paper)*
MS	Multiple sclerosis
NF-κB	Nuclear factor kappa B
NMDA	N-Methyl-D-aspartate
NO	Nitric oxide
NORT	Novel object recognition test
OBX	Olfactory bulbectomy
PD	Parkinson’s disease
PDQ-39	Parkinson’s Disease Questionnaire-39
PFC	Prefrontal cortex
PHQ-15	Patient Health Questionnaire-15
PPARγ	Peroxisome proliferator-activated receptor gamma
PSS-10	Perceived Stress Scale-10
PTS	Post-traumatic stress symptoms
PTSD	Post-traumatic stress disorder
RCT	Randomized controlled trial
REM	Rapid eye movement
RNA	Ribonucleic acid
ROS	Reactive oxygen species
SAD	Social anxiety disorder
SE	Status epilepticus
SKN-1	Skinhead-1 transcription factor (*C. elegans*)
SPST	Simulated public speaking test
SSPS-N	Negative Self-Statements during Public Speaking Scale
STZ	Streptozotocin
THC	Δ^9^-Tetrahydrocannabinol
TNF-α	Tumor necrosis factor alpha
TRPV1	Transient receptor potential vanilloid 1
TRPV2	Transient receptor potential vanilloid 2
TST	Tail suspension test
UPDRS	Unified Parkinson’s Disease Rating Scale
VAMS	Visual Analogue Mood Scale
VCT	Vogel conflict test
WHO	World Health Organization
